# Risk Factor Assessment of Hospice Patients Readmitted within 7 Days of Acute Care Hospital Discharge

**DOI:** 10.3390/geriatrics3010004

**Published:** 2018-01-24

**Authors:** Anthony Wilson, Diana Martins-Welch, Myia Williams, Leanne Tortez, Andrzej Kozikowski, Bridget Earle, Lori Attivissimo, Lisa Rosen, Renee Pekmezaris

**Affiliations:** 1 Northwell Health, Manhasset, NY 11030, USA; aewilsonmd@comcast.net (A.W.); dmartins1@northwell.edu (D.M.-W.); ltortez@gmail.com (L.T.); akozikowski@northwell.edu (A.K.); bearle1@northwell.edu (B.E.); rpekmeza@northwell.edu (R.P.); 2 Hospice Care Network, Woodbury, NY 11797, USA; lattivissi10@northwell.edu; 3 Feinstein Institute for Medical Research, Manhasset, NY 11030, USA; Lisa.m.rosen@hofstra.edu

**Keywords:** hospice, risk factors, palliative care, readmission

## Abstract

Factors surrounding readmission rates for hospice patients within seven days are still relatively unknown. The present study specifically investigates the seven-day readmission rate of patients newly discharged to hospice, and the predictive factors associated with readmission for this population. In a retrospective case-control study, we seek to identify potential predictors by comparing the characteristics of patients discharged to hospice and readmitted within one week to patients who were not readmitted. Cases (n = 46) were patients discharged to home hospice and readmitted to the hospital within seven days. Controls (n = 117) were patients discharged to home hospice and not readmitted to the hospital within seven days. Significant risk factors for readmission within seven days were found to be: age (*p* < 0.01), race (*p* < 0.001), language (*p* < 0.001), and insurance (*p* < 0.001). Further study of these predictors may identify opportunities for interventions that address patient and family concerns that may lead to readmission.

## 1. Introduction

Hospice care seeks to ease the severity of pain that chronically, terminally, or seriously ill patients suffer from, while simultaneously attending to their emotional and spiritual needs [1]. Patients choosing hospice care have chosen to forego curative treatments and medical intervention, including hospitalization, which does not align with the objectives of hospice [2]. Instead, over 95% of hospice care takes place in the home setting, as this is what patients tend to prefer [1]. Hospice care involves visits to the patient or family on an intermittent basis from members of a hospice team (including but not limited to doctors, nurses, social workers, occupational therapists, clergy, or other counselors) to provide care up until the point where the patient remains eligible and wants care. Medicare rules are such that the primary caregiver of the patient is not required to be in the home [1]. Under Medicare Part A, palliative care services are provided to patients with a life expectancy of six months or less who are willing to forgo curative treatments [3]. Ultimately, the goal for patients discharged home to hospice care is to remain at home for symptom management and avoid returning to the hospital.

Previous studies have consistently demonstrated the association between hospice care and reductions in symptom distress, satisfied outcomes for caregivers, and high levels of satisfaction for both patients and their families [3]. For example, Ornstein et al. [4]) found a reduction in the depressive symptoms of spouses of patients who used hospice care than among spouses of patients who were non-hospice users. Miller et al. [5], in a study on the analgesic management of daily pain for dying patients, found that patients enrolled in hospice care reported better pain management than those not enrolled. Similarly, in evaluation studies of family member satisfaction, at least 98% of family members say they would willingly recommend hospice care to others [6,7]. Similarly, recent studies have provided evidence that continuous hospice care reduced the use of hospital-based services such as emergency department visits, intensive care unit stays, and the likelihood of death in the hospital [3,8]. More specifically, research has emphasized the importance of palliative care and hospice services in reducing hospital readmission [3,9,10]. In a study that compared the role of palliative care versus usual care on post-discharge outcomes and hospice use for patients with advanced gastrointestinal (GI) cancers, Paris et al. [11] found supporting evidence for the benefits of combining palliative care and hospice care. Out of a total of 201 patients, 82 received a palliative care consult, and 119 received usual care. At two and four months follow-up, more patients with palliative care consult were receiving hospice care at the time of death than usual care. The researchers concluded that palliative care consultation, when combined with an increase in hospice utilization, decreased the likelihood of patients dying in the hospital, and increased the likelihood of patients dying at home [11]. Data suggest that compared to usual care, a combination of palliative care and hospice reduces health care costs of the sickest patients by providing those patients with the care they need to avoid unnecessary emergency visits, re-hospitalizations and hospital stays [12].

However, despite patient wishes and the goals of hospice care, many patients are re-hospitalized within a week of discharge, and almost 20% of adults 65 years and older are re-hospitalized within 30 days [13]. Previous research has found that 77% of patients utilizing the emergency department in the last 30 days of life were subsequently admitted to the hospital, with 68% of these patients dying in the hospital [14]. Hence, despite an increase in hospice utilization and the benefits of hospice, end of life care continues to be marred by high costs, high-intensity interventions, and multiple transitions of care, including frequent readmissions and hospitalizations [9]. Re-hospitalizations are contradictory to the goals of hospice care. Therefore, it is not surprising that so much emphasis is placed on the combination of hospice and palliative care to improve end of life and reduce re-hospitalizations [9]. For example, the Center for Medicare and Medicaid Services (CMS) now view re-hospitalizations as a core quality measure such that financial penalties are implemented for hospitals with unusually high rates of re-hospitalizations [15].

Hospital admissions and readmissions often contribute to unnecessary suffering and decreased quality of life (QoL) for patients and their family members. In a prospective longitudinal study on patients with advanced cancer and their caregivers, Wright et al. [16] found that patients with cancer who died in the hospital or intensive care unit (ICU) had worse QoL compared with patients who died at home. In addition, the bereaved caregivers of patients who died in the hospital or ICU had an increased risk of developing psychiatric illness compared with patients who died in hospice. Despite the benefits of hospice care, some patients withdraw from hospice care services, while some hospice patients are still hospitalized at the end of life. However, little is known about the patients who are hospitalized after hospice enrollment [17], or the factors predicting readmission for newly discharged patients to hospice care [18].

In an effort to identify predictors of 30-day readmission among older adults newly discharged to hospice care, Goldenheim et al. [18] found that among those readmitted, a significantly lower percentage (25%) were provided with a palliative care consultation, compared to those not readmitted (47.1%), demonstrating that the provision of palliative care consultation is associated with decreased readmissions. Palliative care consultations have also been shown to reduce the length of hospital stays for patients admitted to a medical intensive care unit [19]. Additionally, patients without a participating decision-maker involved in their hospice decision have been found to have approximately three times the risk of readmission within 30 days, compared to those with a decision-maker [19]. Lastly, Goldenheim and colleagues [18] found that patients who had one or more telephone contacts with their primary care physician during the week after discharge had over two times the risk of being readmitted within 30 days, compared to those without.

A similar study by Enguidanos et al. [20] explored factors associated with 30-day hospital readmission among managed care patients receiving a consultation from an inpatient palliative care team. Findings indicated that 10% of patients discharged from the hospital were readmitted within 30 days. Resulting factors associated with increased hospital readmission included being discharged from the hospital with a lack of care in the patient’s home, or to a nursing facility. Also, receiving hospice or home-based palliative care post-discharge was associated with a decreased likelihood of hospital readmission.

Further research is needed to understand the factors surrounding readmission rates for hospice patients. The present study specifically investigates the seven-day readmission rate of patients newly discharged to hospice, and the predictive factors associated with readmission for this population. We seek to identify potential predictors by comparing the characteristics of patients discharged to hospice and readmitted within one week to those patients who were not readmitted.

## 2. Materials and Methods

Electronic medical records of hospice patients were retrospectively reviewed at two academic medical centers at Northwell Health from January 2009 through June 2014. Adult patients eligible for inclusion consisted of those with an acute hospitalization and subsequent discharge to hospice from the acute care hospital, followed by readmission to either the acute care hospital or to inpatient hospice within seven days of discharge. Charts for control group patients were also reviewed (with a ratio of approximately 4:1), which consisted of patients discharged to home but not readmitted within seven days. This was an exploratory study, and the sample was based on feasibility and the availability of resources.

### Statistical Analysis

Descriptive statistics (e.g., means, standard deviations, medians, ranges, and percentages) were used to describe demographic and clinical characteristics of the sample. The chi-square test or Fisher’s exact test, as appropriate, were used to compare categorical predictors of interest between cases and controls. The two-sample *t*-test was used to compare continuous predictors of interest between cases and controls. For predictors that did not meet the standard assumptions of normality needed for a *t*-test, the Mann–Whitney test was used. However, results between the *t*-test and Mann–Whitney were qualitatively similar. Therefore, for ease of interpretation and consistency, only the results from the *t*-test are reported for continuous variables.

## 3. Results

A total of 163 subjects were included in this study, including 46 cases (28.22%) and 117 controls (71.78%). The most frequent hospital diagnosis was cancer (56.4%). Table 1 compares cases and controls on several potential risk factors of interest. There was a significant association between seven-day readmissions and age (*p* < 0.01), race (*p* < 0.001), language (*p* < 0.001), and insurance (*p* < 0.001). Specifically, cases were significantly younger than controls (69.5 vs. 77.0 years), were more likely to be Hispanic (15% vs. 5%), Asian (15% vs. 5%) or of “other” race (13% vs. 2.6%), and were more likely to speak Spanish (13.3% vs. 3.5%) or “other” language (20% vs. 5.3%), and less likely to speak English (67% vs. 91%). Cases were also less likely to have Medicare (8.7% vs. 82.9%) and more likely to have Medicaid (32.6% vs. 4.3%) or other form of insurance, including dual eligibility (45.7% vs. 2.6%). Further, gender, marital status, religion, hospital diagnosis, day of discharge, family support at home, symptoms, and relationship to emergency contact were not significantly associated with seven-day readmission.

## 4. Discussion

Our findings identified four risk factors important for predicting readmission within seven days of acute care hospital discharge, including age, race, language, and insurance status. We found a greater likelihood of seven-day readmission after discharge to hospice for patients who were younger, Hispanic, Asian, or of an “other” race, spoke Spanish or a language other than English, did not have Medicare, and had Medicaid or other form of insurance, including dual eligibility.

Previous studies have also found younger patients to be hospitalized more often than older patients (e.g., Cintron et al., 2003; Hamel et al., 2000; Sharma et al., 2016). This is consistent with the idea that older patients prefer less aggressive care, and are more likely to enroll in hospice earlier [21]. A possible reason for this is because the end of life goals for older and younger patients may differ, which might result in more hospitalizations for younger than older patients. At the end of life, older patients may want to maintain a better quality of life, with lower acceptance of therapy associated with toxicity, be enrolled in hospice earlier than younger patients, and may want to focus less on prolonging their life [17,22]. For example, compared to younger patients, older patients are more likely to have stable preferences for DNR (Do Not Resuscitate) procedures and are likely to change their minds from providing CPR (Cardiopulmonary Resuscitation) to not providing CPR [17,21].

As stated by Hamel et al. [21], it is possible that younger patients are more likely to be hospitalized than older patients because of the “under-treatment” of older patients and the “over-treatment” of younger patients. This is based on the notion that when it comes to less aggressive treatment of older patients, physicians, and by extension family members, have an exaggerated perception of older patient’s desire to avoid aggressive treatment; as a result, there are likely to have an exaggerated perception of the survival disadvantage of older patients [21]. While our analysis does not confirm this, future research should examine the perceptions of family and physicians in terms of treatment for Medicare populations, to determine whether there is an effect on hospitalizations as it relates to older versus younger patients.

Interestingly, with regard to insurance status, our research results contrast with those of recent research indicating that hospice patients with dual eligibility were associated with a lower 30-day readmission rate [23]. Dual eligibility status is an indicator that a patient is of low income, coupled with the possibility of other medical and social factors that are likely to affect health outcomes [24]. Compared with Medicare-only patients, patients who are dual eligibility beneficiaries generally have poorer health, higher prevalence of certain chronic conditions, multiple chronic conditions (e.g., heart failure and COPD (Chronic Obstructive Pulmonary Disease)), mental impairments or functional limitations, higher rates of physical disability, or live in long-term facilities [24,25]. Contrary to Whitney and Chuang [23], previous studies using administrative data have identified that dual-eligible patients have a higher risk of admission and 30-day readmission after hospitalization than both Medicaid and Medicare patients [24]. For example, Bennett and Probst [25] found that dual-eligible patients had higher hospitalization and 30-day readmission rates when compared with Medicare-only beneficiaries. Further, multivariate regressions indicated that patients with dual eligibility who were younger, and had specific chronic conditions, had an increased likelihood of 30-day readmission. A possibility for the need for increased hospitalization might be because patients with dual-eligibility require more medical attention than those with either Medicare or Medicaid; as such, they are more likely to be hospitalized [24].

Our study also found higher readmission rates for patients with Medicaid. It is estimated that at least 61% of adult Medicaid beneficiaries are at increased risk of hospitalizations and readmissions because they have chronic or disabling conditions [26]. Allen et al. [27] found that the unadjusted 30-day all-cause readmission rates for patients in the Medicaid population were significantly higher than for patients who were commercially insured. After adjusting for a wide range of patient factors, the researchers found that patients with Medicaid were 1.32 times more likely to be readmitted for any reason when compared with patients with private insurance. There are many practical barriers as to why patients with Medicaid have higher hospitalization rates than those with other forms of insurance. The length of time for which patients qualify for Medicaid, the possibility of an increase in undocumented immigrants who do not qualify for Medicaid, and the lack of access to inpatient hospice beds for underprivileged or economically disenfranchised patients are all possible reasons for the results found in this present study [28]. Consistent with previous studies, we also found a higher likelihood of seven-day readmission after discharge to hospice for patients who were Hispanic, Asian, or of an “other” race, and spoke Spanish or a language other than English. For example, in a study on Medicare patients with heart failure (HF) and acute myocardial infarction (AMI), Rodriguez et al. [29] found elderly Hispanic patients were more likely to be readmitted for HF and AMI than whites or any other racial group. Similarly, Karliner et al. [30] found that non-English speaking Latino and Chinese patients had a higher risk of readmission than patients who spoke other languages such as Russian and English. 

There are a number of possible reasons for the high admission rates of non-whites and non-English speaking patients. First, errors in communication due to language barriers can result in higher adverse events among non-English speaking patients, which would lead to higher rates of hospitalization [30,31]. Karliner et al. [30] concluded that the higher admission rates for Spanish and Chinese-speaking patients were a result of gaps in communication, which, although present in all patient groups, can be exacerbated by language barriers. Additionally, language barriers are present during hospitalization, but are amplified at discharge. As such, caregivers are unable to understand the needs of the patient for hospice care, which in turn limits the ability of the patient to understand their care plan. Secondly, cultural barriers can also be a possible reason why there are higher rates of hospitalization for Hispanic and other minority patients. For example, culturally, Hispanic patients believe that death is “God’s Will”, and as such, suffering is a natural part of life and death. Since the goal of hospice is symptom and pain management, many Hispanic patients might view hospice practices as anti-ethical, and may opt out of hospice care to return to the hospital during the end of life stages [30].

Another possible reason for high readmission rates among non-English speaking patients could lie in the caregiver’s background, training, and skills, which are often inadequate and negatively impact re-hospitalization rates [31]. When family caregivers are not present, it is rather common for migrant care workers (MCW) to provide full-time support for older family members [31]. Fusco et al. [31], in a study on 506 patients 65 years or older, found that patients who were assisted by MCW were more likely to be re-hospitalized during follow-up. According to Fusco et al. [31], MCW have inadequate health literacy, which can make it difficult to adhere to medication-related instructions. Additionally, cultural barriers such as traditional versus modern medications or treatments beliefs [31,32] of MSW, plus the tendency of the MCW to frequently use hospital care as a response to excessive care responsibilities, and low collaborations with community services, can also result in high re-hospitalization rates [31] in the non-English speaking population.

Results from our study highlighted how patients with a lower socioeconomic status and more complicated health issues are likely to have higher admission rates than those without. A more focused approach targeted at this particular population would help reduce readmission rates. Previous research has shown that an integrative approach (including palliative care and coordinated care team) to end of life care is effective in reducing admission [26]. More specifically, hospitals and even hospice looking to reduce admissions should examine having quality improvement interventions targeted at patients with dual-eligibility or Medicaid [24]. For example, hospitals and hospice could use palliative care interventions and specific drug-eluting stent to reduce admission rates; such practices are underused in patients with dual-eligibility and Medicaid. Furthermore, it is imperative that such interventions are long-term and multi-functional.

Educational programs can also be used as a mechanism for reducing admission rates. Due to the population of patients with high seven-day admission rates, there is a call for interventions that are geared towards realizing and addressing the sensitive values and needs of minority populations [28]. For example, the hospitals and hospice can design outreach programs where patients access care to let them know of alternative services available and the benefits of hospice. Hospice facilities should also reflect the communities that they serve by working in partnership with government and non-profit organizations to make individuals more aware of their services. As recommended by Fusco et al. [31], educating MCW to ascertain their level of health literacy and providing the necessary training to increase health literacy and their ability to provide sufficient care to complex and frail patients can also help reduce re-hospitalizations.

Our study is limited through its retrospective observational design, which does not allow for the determination of causality. We cannot be sure about whether patients’ preferences of care may have changed after hospice enrollment, influencing their decision to be hospitalized. Another limitation is that we could not differentiate between patients who were readmitted to either the acute care hospital or to inpatient hospice within seven days of discharge. However, as one of the first studies to explore factors related to seven-day readmission for newly discharged hospice patients, we highlight the importance of considering factors such as age, race, language, and insurance status, and the role they play in predicting readmissions for this population. Further study of these predictors may identify opportunities for interventions to obviate these readmissions.

Future research should look into examining socioeconomic status and its impact on readmission rates. While we did not take into account socioeconomic status, our results show that individuals who are poverty stricken and are of lower income levels are more likely to have higher admission rates. While there might be a plethora of research on the effects of socioeconomic status, it would be interesting to examine the effects of being a hidden minority (e.g., immigrant) on admission rates. Immigrants might not have access to the healthcare services available, either because they cannot afford such services, or are unaware that such services are available to them. It is also possible that immigrants might have a higher admission rate than native patients since they might be most susceptible to risk factors that predispose them to be admitted. Future research should examine whether admission rates are different among patients who have early-stage cancer versus late-stage cancer, and the length of time at which diagnosis was given. As suggested by Cintro et al. [17], it might be possible that patients with long-term cancer are better able to come to terms psychologically with their illness, and might opt out of prolonging their life when compared to patients with early-stage cancer or recent diagnosis.

## Figures and Tables

**Table 1:** Comparison of Patient Characteristics (*N* = 163). BMI: body mass index.

Variable	Cases (*N* = 46)	Controls (*N* = 117)	*p* Value
Age	69.48 ± 17.68	77.03 ± 13.64	0.0041
BMI	23.55 ± 5.59	23.18 ± 5.97	0.7233
Length of Stay (index hospitalization)	13.52 ± 10.24	10.72 ± 8.70	0.0805
Gender			0.9242
Male	21 (45.65)	52 (44.83)	
Female	25 (54.35)	64 (55.17)	
Race			0.0008
White	16 (34.78)	59 (50.43)	
Black	10 (21.74)	43 (36.75)	
Hispanic	7 (15.22)	6 (5.13)	
Asian	7 (15.22)	6 (5.13)	
Other	6 (13.04)	3 (2.56)	
Marital Status			0.7474
Married	23 (51.11)	52 (44.44)	
Widowed	16 (35.56)	47 (40.17)	
Single/Other	6 (13.33)	18 (15.38)	
Language			0.0007
English	30 (66.67)	104 (91.23)	
Spanish	6 (13.33)	4 (3.51)	
Other	9 (20.00)	6 (5.26)	
Religion			0.1602
Catholic	12 (29.27)	49 (42.61)	
Protestant	13 (31.71)	31 (26.96)	
Jewish	5 (12.20)	20 (17.39)	
Other	8 (19.51)	13 (11.30)	
None	3 (7.32)	2 (1.74)	
Insurance			<0.0001
Medicare Alone	4 (8.70)	97 (82.91)	
Medicaid	15 (32.61)	5 (4.27)	
Private	6 (13.04)	12 (10.26)	
Other (dual eligible, etc.)	21 (45.65)	3 (2.56)	
Hospital Diagnosis			0.5672
Cancer	30 (65.22)	62 (52.99)	
CHF (Chronic Heart Failure)/CAD (Coronary Artery Disease)	5 (10.87)	18 (15.38)	
Dementia	4 (8.70)	14 (11.97)	
Other (i.e., COPD (Chronic Obstructive Pulmonary Disease) and ESRD End Stage Renal Disease)	7 (15.22)	23 (19.66)	
Discharge Day			0.1478
Sunday	2 (4.35)	7 (6.03)	
Monday	7 (15.22)	11 (9.48)	
Tuesday	8 (17.39)	23 (19.83)	
Wednesday	6 (13.04)	28 (24.14)	
Thursday	11 (23.91)	10 (8.62)	
Friday	8 (17.39)	25 (21.55)	
Saturday	4 (8.70)	12 (10.34)	
Family Support at Home			0.1653
Yes	39 (95.12)	116 (99.15)	
No	2 (4.88)	1 (0.85)	
Symptoms			0.3476
Pain	7 (17.07)	16 (16.49)	
Dyspnea	10 (24.39)	12 (12.37)	
All others	21 (51.22)	60 (61.86)	
None	3 (7.32)	9 (9.28)	
Emergency Contact Relationship			0.0743
Spouse	17 (40.48)	33 (28.21)	
Child	23 (54.76)	63 (53.85)	
Other (i.e., family, friend, self)	2 (4.76)	21 (17.95)	

Continuous variables are presented as mean ± SD; Categorical variables are presented as n (%).

## Abbreviations

BMI	Body Mass Index
CAD	Coronary Artery Disease
CHF	Congestive Heart Failure
COPD	Chronic Obstructive Pulmonary Disease
CPR	Cardiopulmonary Resuscitation
DNR	Do Not Resuscitate
ESRD	End Stage Renal Disease
